# IL-1α mediates cellular cross-talk in the airway epithelial mesenchymal trophic unit

**DOI:** 10.1080/21688370.2016.1206378

**Published:** 2016-06-28

**Authors:** Alison R. Hill, Jessica E. Donaldson, Cornelia Blume, Natalie Smithers, Liku Tezera, Kamran Tariq, Patrick Dennison, Hitasha Rupani, Matthew J. Edwards, Peter H. Howarth, Christopher Grainge, Donna E. Davies, Emily J. Swindle

**Affiliations:** a Clinical and Experimental Sciences, Faculty of Medicine, University of Southampton, University Hospital Southampton, Southampton, UK; b NIHR Southampton Respiratory Biomedical Research Unit, University Hospital Southampton, Southampton, UK; c Novartis Institutes for BioMedical Research, Horsham, UK; d Institute for Life Sciences, Highfield Campus, University of Southampton, Southampton, UK

**Keywords:** cross-talk, epithelial cells, fibroblasts, *in vitro* models of the airway, viral infection

## Abstract

The bronchial epithelium and underlying fibroblasts form an epithelial mesenchymal trophic unit (EMTU) which controls the airway microenvironment. We hypothesized that cell-cell communication within the EMTU propagates and amplifies the innate immune response to respiratory viral infections.

EMTU co-culture models incorporating polarized (16HBE14o-) or differentiated primary human bronchial epithelial cells (HBECs) and fibroblasts were challenged with double-stranded RNA (dsRNA) or rhinovirus.

In the polarized EMTU model, dsRNA affected ionic but not macromolecular permeability or cell viability. Compared with epithelial monocultures, dsRNA-stimulated pro-inflammatory mediator release was synergistically enhanced in the basolateral compartment of the EMTU model, with the exception of IL-1α which was unaffected by the presence of fibroblasts. Blockade of IL-1 signaling with IL-1 receptor antagonist (IL-1Ra) completely abrogated dsRNA-induced basolateral release of mediators except CXCL10. Fibroblasts were the main responders to epithelial-derived IL-1 since exogenous IL-1α induced pro-inflammatory mediator release from fibroblast but not epithelial monocultures. Our findings were confirmed in a differentiated EMTU model where rhinovirus infection of primary HBECs and fibroblasts resulted in synergistic induction of basolateral IL-6 that was significantly abrogated by IL-1Ra. This study provides the first direct evidence of integrated IL-1 signaling within the EMTU to propagate inflammatory responses to viral infection.

## Introduction

The structural cells of the conducting airways control the tissue microenvironment and are critical in the maintenance of homeostasis. Central to this is the bronchial epithelium which forms a protective barrier against the external environment, with functions including secretion of a protective layer of mucus, control of paracellular permeability and production of immunomodulatory growth factors and cytokines.^1^ Below the epithelium, the attenuated fibroblast sheath directs immune responses and it has been proposed that these cells work together as an epithelial mesenchymal-trophic unit (EMTU) to co-ordinate appropriate responses to environmental stimuli.^2^

Evidence of cellular cross-talk has already been demonstrated in simple experiments using epithelial-derived conditioned media or in epithelial-fibroblast co-cultures where fibroblasts respond to epithelial-derived signals to drive inflammatory or remodelling responses. For example, conditioned media from human bronchial epithelial cells (HBECs) subjected to endoplasmic reticulum stress can cause proinflammatory mediator release from human lung fibroblasts (HLFs) via a mechanism involving the alarmin, IL-1α.^3^ In other studies, scrape-wounding of HBECs induced α-smooth muscle actin expression in fibroblasts in a co-culture model via TGFβ.^4^ While several studies have examined cross-talk in response to chemical or mechanical damage to the epithelium, none have examined the effects of human rhinovirus (HRV) infection of the epithelium on the EMTU.

HRV infects the upper airways and causes symptoms of the common cold in healthy adults but in chronic respiratory diseases such as asthma and chronic obstructive pulmonary disease (COPD) it is a major cause of viral-induced exacerbations, causing increased lower respiratory tract symptoms.^5,6^ The bronchial epithelium is the major target for HRV infection and replication in chronic airways disease.^7^ Following *in vitro* stimulation of either monolayer or fully differentiated HBECs with HRV or pathogen associated molecular patterns (PAMPs), such as double stranded RNA (dsRNA), increases in ionic permeability^7,8^ and release of proinflammatory mediators are observed.^6,7,9,10^ A critical role for some of these epithelial-derived mediators on immune cell activation has been demonstrated following incubation of immune cells with epithelial conditioned medium from virus or dsRNA-treated cultures. For example, HRV-dependent epithelial IL-33 causes Th2 cytokine release from T cells and group 2 innate lymphoid cells,^11^ while dsRNA-dependent epithelial-derived thymic stromal lymphopoietin promotes CCL17 production from monocyte-derived dendritic cells^12^ and Th2 cytokine release from mast cells.^13^ HRV also induces HBECs to release growth factors such as amphiregulin, activin A, and vascular endothelial growth factor (VEGF);^14-16^ such conditioned medium can result in VEGF-dependent angiogenesis in endothelial cells^14^ and basic fibroblast growth factor-dependent proliferation of fibroblasts.^16^

A key feature of the epithelial barrier is its polarized structure due to the expression of tight junction proteins, leading to the vectorial release of mediators. This not only allows establishment of chemotactic gradients, required for immune cell recruitment and retention, but also controls signaling to underlying fibroblasts which orchestrate responses within the local tissue microenvironment. Here we investigated, for the first time, the integrated responses to HRV infection of the epithelial barrier in co-culture with fibroblasts. Within this system, the polarized epithelium ensured apical delivery to the epithelium of HRV (or dsRNA), as occurs *in vivo*, and enabled direct assessment of vectorial cytokine signaling. We report that challenge of polarized HBECs with dsRNA results in enhanced release of fibroblast-derived proinflammatory mediators in the EMTU model. Furthermore, blockade of IL-1 signaling revealed a key role for basolateral IL-1α release in mediating epithelial-fibroblast cross-talk. These observations of direct epithelial-mesenchymal signaling via IL-1α were confirmed utilizing fully differentiated primary HBECs infected with HRV and in co-culture with fibroblasts.

## Materials and methods

A full description of the methods can be found in the online supplement.

### Cell culture

The human bronchial epithelial (16HBE14o^−^) and fibroblast (MRC5) cell lines used in this study were a gift from Professor D. C. Grunert (San Francisco, USA) and from the European Collection of Authenticated Cell Cultures (ECACC) respectively. Normal primary HBECs were obtained by epithelial brushing using fiberoptic bronchoscopy. All procedures were approved by the Southampton and South West Hampshire Research Ethics Committee (Rec codes 13/SC/0182, 09/H0504/109 and 10/H0504/2) and were undertaken following written informed consent.

### Establishment and challenge of the EMTU co-culture models

For the polarized EMTU model, fibroblasts (MRC5) were seeded onto the basolateral surface of an inverted Transwell® insert and incubated for 2h at 37°C before the addition of 16HBE cells into the apical compartment. Co-cultures were placed into 24-well plates containing 16HBE medium and cultured for 5 d. On day 6, cultures were challenged apically with 1 µg/ml synthetic dsRNA (polyinosinic:polycytidylic acid (poly(I:C)); Invivogen); this concentration had minimal effects on cell viability (Fig. S1A-C). Where required, 16HBE or MRC5 monocultures were similarly treated.

For the primary differentiated EMTU co-culture model, fibroblasts (MRC5) were seeded onto the basolateral surface of inverted Transwell®inserts containing primary fully differentiated air-liquid interface (ALI) (21 day) cultures as previously described.^17^ The primary EMTU models were infected apically with HRV16 for 6h at 33°C, then the apical surface was washed (3X, HBSS) before culturing at 37°C. Twenty four hours post-infection the apical secretions (200 µl) were harvested by washing with HBSS and the basolateral (500 µl) supernatants collected. Controls of UV-irradiated HRV16 (1200mJ/cm^2^ on ice for 50min) were included in all experiments. The viral titer of cell-free supernatants was determined by TCID_50_ assay.^18,19^

For IL-1 blocking experiments, cultures were pre-incubated with IL-1 receptor antagonist (IL-1Ra; 500ng/ml, R&D systems) apically and/or basolaterally for 1h prior to challenge.

MRC5 and 16HBE monocultures were challenged with human recombinant IL-1α (Miltenyi Biotec,) apically (10 ng/ml) and basolaterally (1 ng/ml).

### Epithelial permeability

Ionic permeability was measured as transepithelial electrical resistance (TER) using chopstick electrodes with an EVOM voltohmeter (World Precision Instruments, Aston, UK). Data are expressed as ohms.cm^2^ and have been corrected for the resistance of an empty Transwell®. Macromolecular permeability was measured 3 and 21 hours after dsRNA challenge by adding FITC-labeled dextran to the apical compartment of co-cultures; FITC-dextran flux into the basolateral compartment was quantified 3h later by spectrofluorometry.

### Detection of cytokines and chemokines

Cell-free supernatants were assayed for IL-1α, IL-1β and IL-1Ra using a Luminex®multiplex assay according to the manufacturer's instructions (R&D systems). IL-6, CXCL8, CXCL10, GM-CSF and IL-1α were determined by ELISA according to the manufacturer's protocol (R&D Systems).

### Statistical analysis

Normality of distribution was assessed using the Shapiro-Wilk test (Sigma-Plot version 12.5, Systat Software) and the appropriate parametric or non-parametric tests used. Results are expressed as means ± SD or as box plots representing the median with 25% and 75% interquartiles and whiskers representing minimum and maximum values, as appropriate. All data were analyzed using Prism (GraphPad, CA, USA). *P* < 0.05 were considered significant.

## Results

### DsRNA increases ionic permeability but not macromolecular permeability in the polarized EMTU model

**Figure 1.**
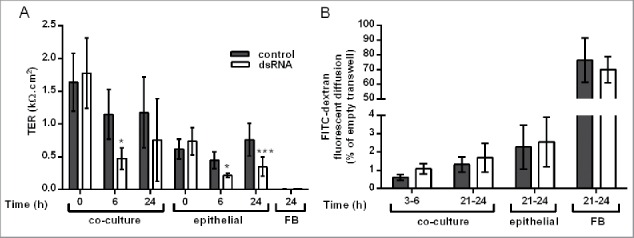
Effect of double-stranded RNA (dsRNA) on epithelial barrier function in the polarized epithelial mesenchymal trophic unit (EMTU) co-culture model. The EMTU co-culture model or HBEC or fibroblast monoculture controls were challenged with poly(I:C) (1μg/ml) and ionic or macromolecular permeability determined by transepithelial resistance (TER) measurements (A) or FITC-dextran diffusion (B) respectively. Results are means ± SD, n = 7 (A) and n = 3–5 (B). *P ≤ 0.05, ***P ≤ 0 .001 compared to unstimulated controls (2-way ANOVA with Bonferroni correction).

Compared to equivalent HBEC monocultures, ionic permeability at baseline was significantly lower in the polarized EMTU model as measured by an increase in TER (Fig. 1A *P* ≤ 0 .05). The polarized EMTU model was stimulated with dsRNA (poly(I:C)), a molecular pattern associated with viral replication,^20^ at a concentration (1 µg/ml) that induced significant effects on ionic permeability and cytokine release with minimal effects on cell viability in HBEC monocultures (Fig. S1). DsRNA increased ionic permeability of either HBEC monocultures or the polarized EMTU model, with a significant decrease in TER by 6h (Fig. 1A). This increase in permeability was sustained 24h after dsRNA stimulation in HBEC monocultures, but partially recovered in the EMTU model. Macromolecular permeability of the epithelium was not significantly affected by co-culture with fibroblasts or following challenge with dsRNA (Fig. 1B). These data suggest that even after dsRNA treatment, epithelial polarization is maintained in the polarized EMTU model.

### DsRNA induces polarized release of proinflammatory mediators which is enhanced in the basolateral compartment

**Figure 2.**
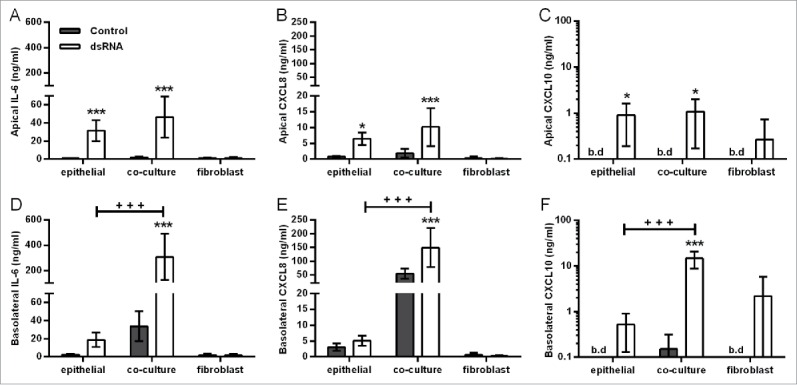
Effect of double-stranded RNA (dsRNA) on proinflammatory mediator release in the polarized epithelial mesenchymal trophic unit (EMTU) co-culture model. Apical (A-C) and basolateral (D-F) cell-free supernatants were harvested from the EMTU co-culture model or human bronchial epithelial cell (HBEC) and fibroblast monocultures 24h after challenge with poly(I:C) (1 µg/ml) and assayed for IL-6 (A,D), CXCL8 (B,E), and CXCL10 (C,F) by ELISA. Results are means ± SD, n = 3–5. *P ≤ 0.05, and ***P ≤ 0.001 for comparison between control and poly(I:C)-stimulated cultures and ^+++^*P* ≤ 0 .001 for comparison with HBEC monocultures and EMTU co-culture model (2-way ANOVA with Bonferroni correction). b.d. indicates levels below the detection limit of the assay.

Consistent with the restricted movement of macromolecules across the epithelial barrier, dsRNA induced vectorial proinflammatory mediator release in the polarized EMTU model. In the apical compartment, dsRNA induced significant increases in IL-6, CXCL8, and CXCL10 release which was comparable with HBEC monocultures (Fig. 2A–C). In contrast, in the basolateral compartment, dsRNA-stimulated cytokine levels were synergistically enhanced compared to dsRNA-stimulated HBEC monocultures (Fig. 2D–F, Fig. S2). At the concentration of dsRNA tested (1 µg/ml), fibroblast monocultures were unresponsive to stimulation (Fig. 2A–F). Taken together, these data suggest that epithelial-fibroblast cross-talk is occurring within the EMTU model.

**Figure 3.**
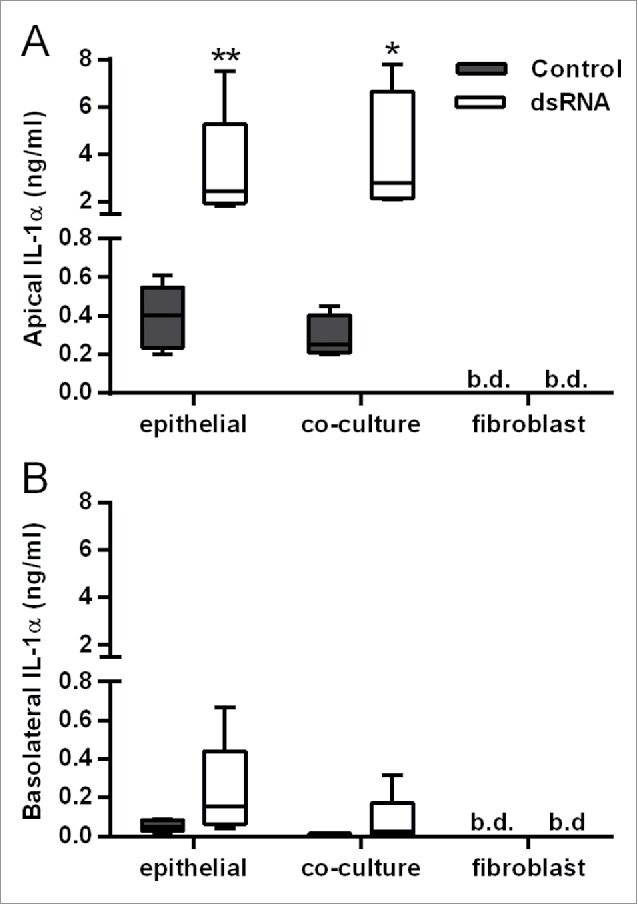
Comparison of IL-1α release from double-stranded RNA (dsRNA)-stimulated human bronchial epithelial cell (HBEC) and fibroblast monocultures with the polarized epithelial mesenchymal trophic unit (EMTU) co-culture model. Apical (A) and basolateral (B) cell-free supernatants were harvested 24 h after challenge with poly(I:C) (1 µg/ml) and assayed for IL-1α and IL-1β by Luminex®. Results for IL-1α release are shown as box plots representing the median with 25% and 75% interquartiles, and whiskers representing minimum and maximum values, n = 3–5. *P ≤ 0.05, **P ≤ 0.01 for comparison between control and poly(I:C) stimulated cultures (Mann-Whitney U test). b.d. indicates levels below the detection limit of the assay. IL-1β was below the level of detection of the assay.

In contrast with IL-6, CXCL8, GM-CSF and CXCL10 release, the polarity of dsRNA-dependent IL-1α release was mainly apical (Fig. 3A–B), even when corrected for differences in volume between the apical and basolateral compartments (data not shown), and was comparable between HBEC monocultures and the polarized EMTU model. No IL-1β was detected. Since IL-1α levels were similar in cultures containing HBECs alone this strongly suggests that HBECs are the primary source of IL-1α following dsRNA stimulation.

### IL-1 mediates dsRNA-dependent proinflammatory responses

**Figure 4.**
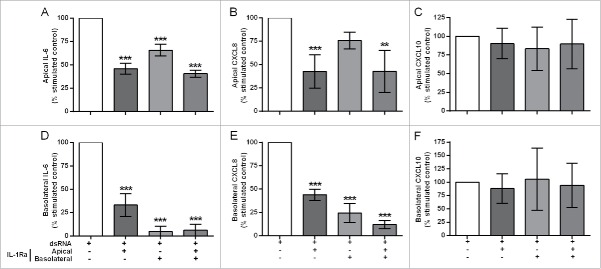
The effect of IL-1R antagonism on double-stranded RNA (dsRNA)-induced cytokine and chemokine release in the polarized epithelial mesenchymal trophic unit (EMTU) co-culture model. The EMTU co-culture model was cultured in the absence or presence of IL-1Ra (500 ng/ml) applied either apically, basolaterally or both for 1h prior to stimulation with poly(I:C) (1 µg/ml). Apical (A-C) and basolateral (D-F) cell-free supernatants were harvested 24 h after stimulation and assayed for IL-6 (A, D), CXCL8 (B, E), and CXCL10 (C, F) by ELISA. To investigate the effects of IL-1Ra on dsRNA-dependent responses, control mediator levels were subtracted from stimulated levels and expressed as a percentage of the response to dsRNA. Results are mean responses compared to the poly(I:C)-induced response in the absence of IL-1Ra (100%) ± SD, n = 3–6. **P ≤ 0.01, ***P ≤ 0.001 for comparison between poly(I:C)-stimulated cultures in the absence or presence of IL-1Ra (one-way ANOVA with Bonferroni correction).

IL-1 has previously been shown to drive autocrine mediator release in epithelial^10^ or fibroblast^3^ monocultures. To test whether epithelial-derived IL-1α was responsible for augmenting responses in the EMTU model, we used IL-1 receptor antagonist (IL-Ra). In unstimulated cultures, IL-1Ra caused a small decrease in constitutive proinflammatory mediator release (Fig. S3). In dsRNA-stimulated co-cultures, pre-incubation with IL-1Ra significantly reduced dsRNA-induced IL-6, CXCL8 and GM-CSF release (Fig. 4, Fig. S4 and Table S1). For apical cytokine release, IL-1Ra only partially reduced dsRNA-dependent IL-6 and CXCL8 (Fig. 4A–B) release and was most effective when added apically or to both compartments. For basolateral cytokine release, IL-1Ra had the greatest effect when added basolaterally or to both compartments with complete abrogation of dsRNA-dependent IL-6, CXCL8 and GM-CSF (Fig. 4D–E & Fig. S4). The partial inhibitory effect of IL-1Ra when added apically could be explained by a small (0.1–1%) but significant passage of exogenously applied IL-1Ra to the basolateral compartment regardless of dsRNA stimulation (Fig. S5). Neither apical nor basolateral dsRNA-dependent CXCL10 release was affected by IL-1Ra (Fig. 4C, F). Since IL-1β could not be detected in any cultures, these data suggest that epithelial-derived IL-1α is absolutely required to drive a subset of proinflammatory responses by the underlying fibroblasts.

### Fibroblasts are the main responders to IL-1α

**Figure 5.**
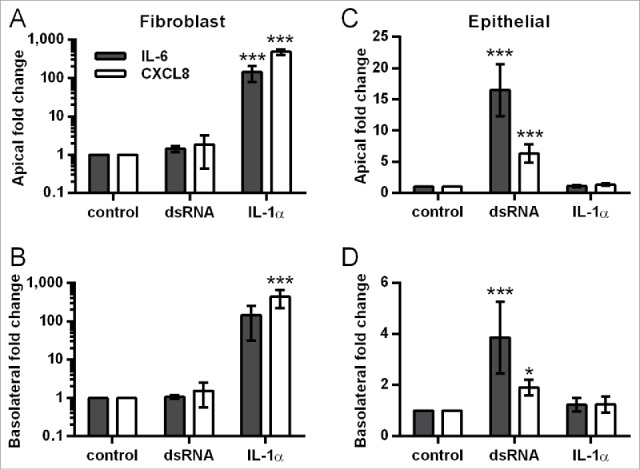
Effect of IL-1α stimulation on IL-6 and CXCL8 release from fibroblast and human bronchial epithelial cell (HBEC) monocultures. Fibroblast (A-B) and HBEC (C-D) monocultures were stimulated with IL-1α either apically (10 ng/ml), basolaterally (1ng/ml) or in combination, or with poly(I:C) (1 µg/ml) as a positive control. After 24 h, cell-free supernatants were assayed for IL-6 and CXCL8 by ELISA. Fold change in mediator release compared to the unstimulated control was calculated for each experiment. Results are mean fold changes ± SD, n = 4–5. *P ≤ 0.05, ***P ≤ 0.001 compared to untreated control (2-way ANOVA with Bonferroni correction).

To investigate the direct effect of IL-1α on the different cell types, HBEC and fibroblast monocultures were directly stimulated with IL-1α at concentrations similar to those measured apically (10 ng/ml) or basolaterally (1 ng/ml) following dsRNA challenge (See Fig. 3). In fibroblast monocultures IL-1α significantly induced IL-6 and CXCL8 release (Fig. 5A–B and Table S2). In HBEC monocultures, IL-1α responses were low relative to those observed in the fibroblasts (Fig. 5C–D & Table S2) suggesting that within the polarized EMTU model, fibroblasts are the main responders to dsRNA-induced IL-1α.

### A role for IL-1alpha in epithelial-fibroblast signaling in response to rhinovirus infection in a primary EMTU co-culture model

**Figure 6.**
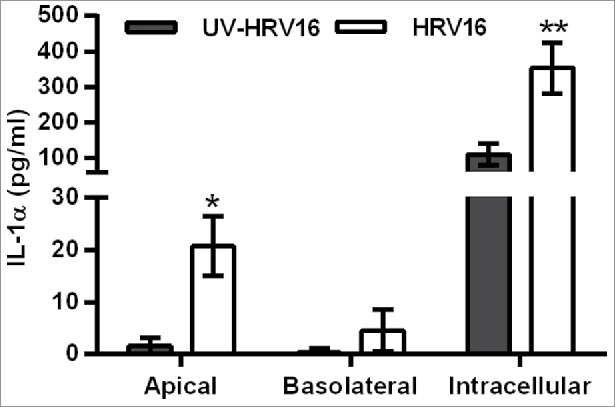
Increased extracellular and intracellular IL-1α release from human bronchial epithelial cell (HBEC) monocultures infected with human rhinovirus (HRV)16. ALI monocultures were infected apically with HRV16 (MOI = 2) or UV-HRV16 as a negative control. After 24h, apical and basolateral supernatants were removed and the remaining cells went through 3 cycles of freeze/thaw before cell-free supernatants were assayed for IL-1α by ELISA. Results are means ± range, n = 5. *P ≤ 0.05, **P ≤ 0.01 compared to UV-HRV16 control (ANOVA with Bonferroni correction).

As HBECs are the primary source of IL-1α, we initially characterized the response of fully differentiated primary HBECs to HRV16 infection. Similar to the dsRNA-challenged polarized EMTU model, HRV16 infection induced IL-1α release from HBEC ALIs which was higher in the apical compared to the basolateral compartment (Fig. 6). IL-1α was also detected intracellularly and was significantly increased following HRV16 infection. Of note, the amount of intracellular IL-1α production was 50-100X greater than that detected extracellularly following HRV infection.

**Figure 7.**
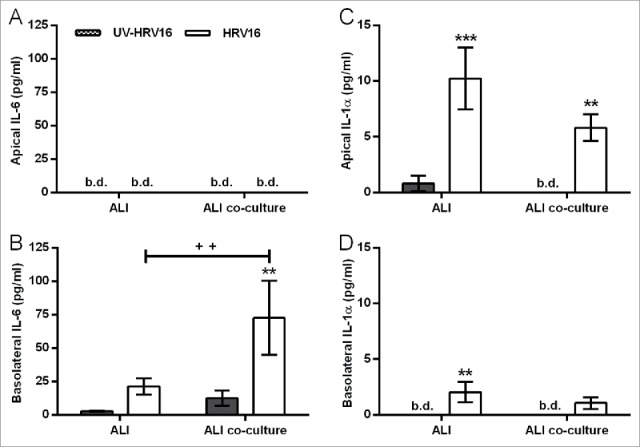
Increased human rhinovirus (HRV)16-induced IL-6 and IL-1α release from the primary differentiated epithelial mesenchymal trophic unit (EMTU) co-culture model compared to air-liquid interface (ALI) monocultures. ALI mono- or co-cultures with fibroblasts were infected apically with human rhinovirus (HRV)16 (MOI = 2) or UV-HRV16 as a negative control. After 24 h, apical (A, C) and basolateral (B, D) cell-free supernatants were assayed for IL-6 (A-B) or IL-1α (C-D). Results are means ± SD, 3 separate experiments from one epithelial cell donor and are representative of 3 donors. **P ≤ 0.01, ***P ≤ 0.001 compared to UV-HRV16 control and ^++^*P* ≤ 0.01 comparing HRV16-treated mono- and co-cultures (2-way ANOVA with Bonferroni correction). b.d. indicates levels below the detection limit of the assay.

**Figure 8.**
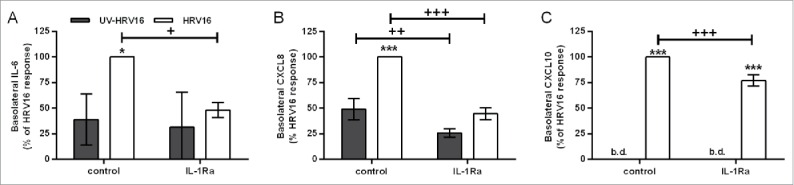
Role for IL-1α in human rhinovirus (HRV)16-induced proinflammatory responses in the primary differentiated epithelial mesenchymal trophic unit (EMTU) co-culture model. Co-cultures were treated with IL-1Ra (500 ng/ml) basolaterally for 1 h prior to HRV16 (MOI = 2) or UV-HRV16 as a negative control. After 24 h, cell-free supernatants were assayed for IL-6 (A), CXCL8 (B), and CXCL10 (C) by ELISA. To examine the effect of IL-1Ra on HRV16-induced cytokine release, cytokine levels are expressed as % of HRV16-induced control response (100%). Results are means ± SD, n = 3 separate epithelial cell donors. ***P ≤ 0.001 compared to UV-HRV16 control and ^+^*P* ≤ 0.05, ^++^*P* ≤ 0.01 or ^+++^*P* ≤ 0.01 comparing control and IL-1Ra-treated cultures (2-way ANOVA with Bonferroni correction). b.d. indicates levels below the detection limit of the assay.

In either HBEC mono- or co-cultures with fibroblasts, HRV16 infection resulted in polarized release of mediators. HRV16-dependent basolateral IL-6 release was significantly augmented in the primary EMTU co-culture model compared to HBEC monocultures (Fig. 7A–B). This enhancement was not due to differences in viral replication (median TCID_50_ of 17.6×10^6^/ml in both primary HBEC monocultures and differentiated EMTU model). As observed with dsRNA, HRV16-dependent IL-1α release was higher in the apical compartment and levels were comparable in both the primary EMTU co-culture model and HBEC monocultures (Fig. 7C–D). HRV16-dependent IL-1β release was not detected. The importance of IL-1 in epithelial-fibroblast cross-talk was confirmed by blocking IL-1 signaling using IL-1Ra. This significantly reduced basolateral HRV16-dependent IL-6 and CXCL8 release (Fig. 8A–B) to levels comparable to the non-replicating UV-irradiated HRV control. CXCL10 release was only modestly reduced (Fig. 8C) and viral replication was unaffected (median TCID_50_ of 17.6×10^6^/ml in both control and IL-1Ra-treated cultures). Together these data demonstrate an essential role for IL-1α in mediating paracrine proinflammatory signaling following viral infection of primary differentiated epithelium.

## Discussion

Although cell-cell communication is essential for normal function of all tissues, the relationship between structural organization and function is not addressed in most *in vitro* studies. Here we examined this relationship using an integrated co-culture system in which fully differentiated (or polarized HBECs) were apically challenged with HRV (or dsRNA) and demonstrated clear evidence of a synergistic interaction between the infected bronchial epithelium and fibroblasts. This interaction was mediated, in part, by epithelial-derived IL-1α which drives a marked proinflammatory response from the underlying fibroblasts. To our knowledge this is the first study to demonstrate direct epithelial-fibroblast cross-talk in response to HRV infection or dsRNA and it highlights the importance of epithelial barrier function and integrity.

An advantage of the EMTU models is the ability to investigate polarized epithelial function which is essential for development of chemotactic gradients for immune cell trafficking and/or retention. In contrast with previous studies using epithelial monocultures, where HRV (or dsRNA) increased both ionic and macromolecular permeability,^7,8,21,22^ we show that only ionic permeability is affected in the EMTU models. Consistent with the absence of any effects on paracellular permeability, apical challenge of the epithelium with HRV or dsRNA resulted in polarized inflammatory mediator release. Most notably, a synergistic enhancement in the basolateral compartment of the EMTU models suggests a coordinated response to viral infection. This was observed in both the polarized and primary EMTU models but the magnitude of the enhanced responses was different between cultures. The less robust response observed in the primary EMTU model may be due to the use of HRV instead of dsRNA. For a response to HRV, the virus first needs to infect the epithelial cells and replicate to generate dsRNA, in contrast with the bolus treatment with exogenously added dsRNA. Furthermore, the fully differentiated epithelial culture has a protective mucus layer which may reduce accessibility of the epithelial surface to the virus and, even if the HRV reaches the cell surface, differentiated epithelial cells are less susceptible to infection than basal cells.^23^ Irrespective of the differences in the magnitude of response, synergistic enhancements in basolateral mediator release in both models suggest cross-talk between epithelial cells and fibroblasts following viral infection. This adds to previous studies where influenza virus infection enhanced mediator release in alveolar epithelial cell and fibroblast co-cultures, however polarized responses were not examined.^24^ The ability of fibroblasts to respond to and amplify signals from a virally-infected epithelium reflects their role as sentinels of the immune system.^2,25,26^

In the EMTU models we determined a key role for epithelial-derived IL-1α in mediating cellular cross-talk and amplifying innate immune responses following viral stimulation. IL-1α is constitutively expressed in the cytoplasm of cells and is released in a mature form following necrotic cell death, however it can also be released in the absence of cell death.^10,27-29^ While we found no evidence of epithelial cell death in the co-culture model following dsRNA (Fig. S1C), we observed approximately 10% cell death in HRV-infected ALI cultures. However we also observed upregulation of intracellular IL-1α in HBECs following exposure to HRV or dsRNA suggesting intracellular IL-1α protein is induced by viral challenge and may be actively released, as reported previously.^10^ We also concluded that the IL-1α was epithelial-derived since it was detected equivalently in HBEC mono- and co-cultures but not in fibroblast monocultures. This is consistent with immunohistochemical staining of bronchial tissue showing that the epithelium is a major site of IL-1α expression,^3^ with localization toward the apical surface of the epithelium.

The polarized nature of the models also gave us the opportunity to investigate the importance of apical and basolateral IL-1 signaling. Thus, basolateral application of IL-1Ra was sufficient to completely suppress basolateral release of IL-6, CXCL8 and GM-CSF, but had minimal effect on CXCL10 release. As CXCL10 is strongly induced by Type I and III interferons, it is of considerable interest that this anti-viral response can be separated from the IL-1α mediated proinflammatory response. In contrast with its potency in the basolateral compartment, apical application of IL-1Ra was less effective with only a partial suppression of mediator release. Although both IL-1α and IL-1β can be inhibited by the use of IL-1Ra,^30^ in our system it is likely that IL-1Ra primarily blocks IL-1α signaling as we could not detect IL-1β in the EMTU co-culture models. IL-1β has previously been detected from primary HBEC monolayer cultures following viral infection,^10,31,32^ however we could not detect it in our models using differentiated HBEC cultures. This may be due to use of undifferentiated cells versus polarized or fully differentiated cultures. Our data suggest that in response to dsRNA or HRV, epithelial cells release IL-1α basolaterally and that this is required to drive IL-6, CXCL8 and GM-CSF release from fibroblasts. Consistent with this, we showed that the fibroblasts were highly sensitive to direct stimulation with IL-1α. These results are consistent with previous findings that IL-1α present in conditioned medium from damaged epithelial cells induces IL-6 and CXCL8 production from fibroblasts.^3,33^

Given the relatively high levels of apically released IL-1α, it was surprising that the low levels of basolateral IL-1α measured in the EMTU co-culture models were not only sufficient, but essential, for dsRNA-induced proinflammatory mediator release in this compartment. This may be explained by the close proximity of the fibroblasts to the basolateral surface of the epithelium resulting in high localized concentrations of IL-1α. Also IL-1R1 is highly expressed by fibroblasts^3^ suggesting that they are highly sensitive to activation, even at low concentrations of IL-1α. Furthermore IL-6 is known to act as an autocrine factor that can drive its own release,^34^ thus IL-1α may be a trigger for this effect. In contrast to the marked sensitivity of fibroblasts to exogenous or paracrine IL-1α, HBECs were relatively unresponsive to direct IL-1α stimulation. Thus, we observed little response using a concentration similar to that measured in the cell-free supernatants of challenged cultures; however, at higher concentrations of IL-1α, IL-6 production could be observed (data not shown). Furthermore, when HBEC monocultures were challenged with dsRNA in the presence of IL-1Ra, partial inhibition of dsRNA-dependent cytokine release was observed, similar to findings with HRV-infected HBECs.^10^ In such a complex antiviral response, it is possible that other factors synergize with IL-1α to promote an epithelial inflammatory response.

Although out of the scope of the current study, the high levels of IL-1α in the apical compartment are of considerable interest as they have the potential to amplify local innate and adaptive immunity through direct activation or enhancement of luminal immune cell functions. Macrophages are the first line of cellular defense against invading pathogens and the IL-1α-IL-1RI pathway has been identified as a key driver of inflammatory cytokine and chemokine activation after adenovirus infection.^35^ However, direct evidence for IL-1α-mediated cross talk with infected epithelium has not been investigated. The human monocytic cell line, THP-1, expresses IL-1R1 and alveolar macrophages have reduced LPS-dependent CXCL8 release in the presence of IL-1Ra.^3,36^ Mast cells also respond to IL-1α with enhanced Th2 cytokine production.^37,38^

In conclusion, we provide evidence of direct cellular cross-talk in an integrated model of the EMTU where apical HRV infection or exposure to dsRNA of the epithelium results in the maintenance of polarized responses and drives synergistic basolateral proinflammatory mediator release from underlying fibroblasts. Epithelial-derived IL-1α plays a key role in enhancing proinflammatory but not anti-viral responses of the underlying fibroblasts. In chronic respiratory diseases, such as asthma and COPD, where respiratory viral infections are a major cause of acute exacerbations^6^ targeting IL-1α may suppress airway inflammation while maintaining anti-viral signaling. The IL-1R1 antagonist anakinra is already FDA-approved^39^ and clinical trials have shown its effectiveness in inflammatory diseases^40^ and LPS-induced airway inflammation in healthy volunteers without adverse effects.^41^

## Supplementary Material

KTIB_S_1206378.docx

## Abbreviations

16HBE: the 16HBE14o^−^ human bronchial epithelial cell line
ALI: air-liquid interface
BEC: bronchial epithelial cell
dsRNA: double-stranded RNA
EMTU: epithelial-mesenchymal trophic unit
HBEC: human bronchial epithelial cell
HLF: human lung fibroblast
HRV: human rhinovirus
IL-1R1: IL-1 receptor
IL-R2: IL-1 decoy receptor
IL-1Ra: IL-1 receptor antagonist
MOI: multiplicity of infection
poly(I:C): polyinosinic:polycytidylic acid
TCID_50_: tissue culture infective dose resulting in 50% death
TER: transepithelial electrical resistance
