# Supplementary material for: IL-1α mediates cellular cross-talk in the airway epithelial mesenchymal trophic unit
Source: Tissue Barriers. 2016 Jun 28;4(3):e1206378. doi: 10.1080/21688370.2016.1206378 (PMC4993579; doi:10.1080/21688370.2016.1206378)
Supplement: KTIB_S_1206378.docx [file ktib-04-03-1206378-s001.docx]

**SUPPLEMENTARY MATERIAL**

**IL-1α mediates cellular cross-talk in the airway epithelial mesenchymal trophic unit**

Alison R Hill^a^, Jessica E Donaldson^a^, Cornelia Blume^a^, Natalie Smithers^a^, Liku Tezera^a^, Kamran Tariq^b^, Patrick Dennison†, Hitasha Rupani^a, b^, Matthew J Edwards^d^, Peter H Howarth^a, b^ Christopher Grainge^a¶^, Donna E Davies^a, b, c^ and Emily J Swindle^a, b, c*^.

^a^Clinical and Experimental Sciences, Faculty of Medicine, University of Southampton, University Hospital Southampton, Southampton, UK; ^b^NIHR Southampton Respiratory Biomedical Research Unit, University Hospital Southampton, Southampton, UK; ^c^Institute for Life Sciences, Highfield Campus, University of Southampton, Southampton, UK; ^d^Novartis Institutes for BioMedical Research, Horsham, UK.

***Corresponding author and requests for reprints:**

Dr Emily J Swindle,

Clinical and Experimental Sciences,

Henry Wellcome Laboratories

Level F, South Academic Block, Mailpoint 810, Faculty of Medicine,

University Hospital Southampton, Southampton SO16 6YD, UK.

Tel.: +44(0)23 8079 6736;

Fax.: +44(0)23 8051 1761;

E-mail: [E.J.Swindle@soton.ac.uk](mailto:E.J.Swindle@soton.ac.uk)

¶,present address: School of Medicine and Public Health, The University of Newcastle, Australia

**SUPPLEMENTARY METHODS**

**Cell culture.** The human bronchial epithelial cell line, 16HBE14o^-^, (16HBE, a gift from Professor D. C. Grunert, San Francisco, USA), was maintained in MEM with Glutamax and supplemented with 10% heat-inactivated FBS and penicillin (50IU/ml)/streptomycin (20μg/ml). The fibroblast cell line, MRC5, was maintained in DMEM supplemented with 10% FBS, penicillin (50IU/ml)/streptomycin (20μg/ml), ʟ-glutamine (1%), non-essential amino acids (1%) and sodium pyruvate (1%). All cell culture reagents were supplied by Life Technologies)

**Establishment of the polarized EMTU co-culture model.** Transwell^®^ culture inserts (6.5mm diameter, 0.4µm pore size permeable polyester membrane; Corning, the Netherlands) were used for the culture of epithelial cells (16HBE) and fibroblasts (MRC5). After coating the membrane with collagen I (30µg/ml; Advanced Biomatrix, USA), inserts were inverted and seeded with MRC5 cells (5x10^4^ cells in 50μl medium/Transwell^®^) and incubated in a humidified incubator at 37^o^C, 5% CO_2_ for 2h to allow adherence. Non-adherent cells were gently washed away with HBSS, inserts inverted and placed in 24-well plates containing 16HBE medium (500μl) before 16HBEs cells (1.5x10^5^ cells in 200μl medium/well) were seeded into the apical compartment. Hence 16HBEs were cultured on the apical surface while MRC5 cells were cultured on the basolateral surface of the permeable culture insert. Control cultures of 16HBE and MRC5 cells alone were also established. Cells were cultured for 5 days and media changed every 2-3 days.

**Establishment of the primary differentiated EMTU co-culture model.** Primary fully differentiated ALI cultures were established as previously described [^1^](#_ENREF_1) and at day 21 the underside of the Transwell^®^ was seeded with MRC5 cells as described above. After fibroblast attachment, the inserts were placed in 24-well plates containing BEC basal medium (500µl) (Lonza, Switzerland) containing insulin (5μg/ml), transferrin (5μg/ml) and sodium selenite (5ng/ml) (ITS; Sigma, UK), BSA (0.01%; Sigma, UK) and penicillin (50IU/ml)/streptomycin (20μg/ml) before HRV16 infection the next day.

**Assessment of macromolecular permeability.** Three or 21 hours after apical challenge with dsRNA, FITC-labelled dextran (4kDa or 20kDa, 2mg/ml, Sigma, UK) was added to the apical compartment and incubated for 3h in a humidified atmosphere of 5% CO_2_ at 37^o^C. Samples (50µl) were then removed from the basolateral compartment and quantified by comparison with a FITC-dextran standard curve (2–1000µg/ml) and fluorescence (ex 485nm and em 530nm) determined using a Fluoroskan Ascent FL2.5 plate reader (ThermoFisher, UK). FITC-dextran diffusion was expressed as a percentage of diffusion through an empty Transwell^®^.

**Detection of cytokines and chemokines.** Cell-free supernatants were assayed for IL-1α (detection range; 3 - 2300pg/ml), IL-1β (detection range; 4 – 2700pg/ml) and IL-1Ra (detection range 13 – 9,000pg/ml) using a Luminex^®^ screening assay on a Bio-Rad Bioplex 200 platform according to the manufacturer’s instructions (R&D systems, UK). IL-6 (detection range; 9 – 600pg/ml), CXCL8 (detection range; 31 - 2,000pg/ml), CXCL10 (detection range; 31 - 2,000pg/ml) and GM-CSF (detection range; 6-750pg/ml) were determined by ELISA according to the manufacturer’s protocol (R&D Systems, UK)

**SUPPLEMENTARY REFERENCE**

1. Blume C, Swindle EJ, Dennison P, Jayasekera NP, Dudley S, Monk P, et al. Barrier responses of human bronchial epithelial cells to grass pollen exposure. Eur. Respir. J. 2013; 42:87-97.

**SUPPLEMENTARY FIGURES**

**Supplementary figure 1.** Concentration responses of polarized HBEC monocultures to dsRNA. Polarised HBEC monocultures were apically challenged with poly(I:C) (0.1-10µg/ml). Over 24h. TER was measured at 6 and 24h post-stimulation and expressed as TER relative to the TER value prior to challenge (n=3) (A). After 24h, apical and basolateral cell-free supernatants were assayed for IL-6 (B) and LDH release (C). LDH release was determined by a non-radioactive cytotoxic assay according to manufacturer’s instructions (Promega, Southampton, UK). Treatment of cells with 1% Triton X-100 was used as a positive control for 100% cell lysis and used to construct a standard curve. Spiking the positive control with poly(I:C) did not interfere with the assay. Results are means ± SD, n=3-4. **P*≤0.05, ***P*≤0.01 and ****P*≤0.001 compared to controls (two-way ANOVA with Bonferroni correction).

**Supplementary Figure 2.** Effect of double-stranded RNA (dsRNA) on GM-CSF release in the polarized epithelial mesenchymal trophic unit (EMTU) co-culture model. Apical and basolateral cell-free supernatants were harvested 24h after stimulation of the EMTU co-culture model or HBEC and fibroblast monocultures with poly(I:C) (1µg/ml). Supernatants were assayed for GM-CSF by ELISA. Results are shown as box plots representing the median with 25% and 75% interquartiles, and whiskers representing minimum and maximum values, n=3-5. ***P*≤0.01 for comparison between control and poly(I:C)-stimulated cultures (Kruskal-Wallis test with Dunn’s correction). b.d. indicates levels below the detection limit of the assay.

**Supplementary Figure 3**. Effect of IL-1Ra on constitutive cytokine release in the EMTU co-culture model. HBEC and fibroblast co-cultures were treated with IL-1Ra (500ng/ml) either apically, basolaterally or in combination for 25h before harvesting apical (A-C) and basolateral (D-F) cell-free supernatants for detection of IL-6 (A, D), CXCL8 (B, E) and GM-CSF (C, F) by ELISA. The effects of IL-1Ra were expressed as a % of untreated control for each experiment (see Table 1 for raw data). Results are means ± SD, n=3-6. ***P*≤0.01 compared to untreated controls (one-way ANOVA with Bonferroni correction).

**Supplementary Figure 4** The effect of IL-1R antagonism on double-stranded RNA (dsRNA)-induced GM-CSF release in the polarized epithelial mesenchymal trophic unit (EMTU) co-culture model. The EMTU co-culture model was cultured in the absence or presence of IL-1Ra (500ng/ml) applied either apically, basolaterally or both for 1h prior to stimulation with poly(I:C) (1µg/ml). Apical and basolateral cell-free supernatants were harvested 24h after stimulation and assayed for GM-CSF by ELISA. To investigate the effects of IL-1Ra on dsRNA-dependent responses, control mediator levels were subtracted from stimulated levels and expressed as a percentage of the response to dsRNA. Results are mean responses compared to the poly(I:C)-induced response in the absence of IL-1Ra (100%) ± SD, n=3. **P*≤0.05, for comparison between poly(I:C)-stimulated cultures in the absence or presence of IL-1Ra (one-way ANOVA with Bonferroni correction). b.d. indicates levels below the detection limit of the assay.

**Supplementary Figure 5.** Macromolecular flux of exogenous IL-1Ra from the apical to basolateral compartments in unstimulated and dsRNA-stimulated co-cultures. Exogenous IL-1Ra (500ng/ml) was added to the apical compartment of the polarized EMTU model and incubated for 1h prior to stimulation with poly(I:C) (1μg/ml). Basolateral cell-free supernatants were harvested after 24h for detection of IL-1Ra by Luminex® assay. Results are means ± SD, n=3. **P*≤0.05, ***P*≤0.01 comparing cultures with and without exogenous IL-1Ra in the apical compartment (two-way ANOVA with Bonferroni correction).

Table 1. The effect of IL-1R antagonism on double-stranded RNA (dsRNA)-induced cytokine and chemokine release in the polarized epithelial mesenchymal trophic unit (EMTU) co-culture model. The EMTU co-culture model was cultured in the absence or presence of IL-1Ra (500ng/ml) applied either apically, basolaterally or both for 1h prior to stimulation with poly(I:C) (1µg/ml). Apical and basolateral cell-free supernatants were harvested 24h after stimulation and assayed for IL-6, CXCL8, CXCL10 and GM-CSF by ELISA. Results are means ±SD, n=3-6. b.d. indicates levels below the detection limit of the assay. Raw data for figure 4A-F, supplemental figure 2B and supplemental figure 3A-F.

|  | **IL-6 (ng/ml)** | | | | **CXCL8 (ng/ml)** | | | | **CXCL10 (ng/ml)** | | | | **GM-CSF (pg/ml)** | | | |
| --- | --- | --- | --- | --- | --- | --- | --- | --- | --- | --- | --- | --- | --- | --- | --- | --- |
|  | **Apical** | | **Basolateral** | | **Apical** | | **Basolateral** | | **Apical** | | **Basolateral** | | **Apical** | | **Basolateral** | |
| **dsRNA** | **-** | **+** | **-** | **+** | **-** | **+** | **-** | **+** | **-** | **+** | **-** | **+** | **-** | **+** | **-** | **+** |
| **- IL-1Ra** | 1.8±1.9 | 33.9±28.2 | 25.6±22.3 | 207.7±194.7 | 1.7±0.7 | 9.3±4.1 | 58.8±24.7 | 172±68.5 | b.d. | 0.8±0.5 | 0.1±0.1 | 6.6±3.1 | b.d. | b.d. | 74±87 | 276±105 |
| **+ IL-Ra apical** | 1.3±1.4 | 16.0±14.0 | 24.8±19.5 | 93.9±90.4 | 1.1±0.5 | 4.0±1.4 | 41.8±14.1 | 93.2±38.9 | b.d. | 0.7±0.6 | b.d. | 6.3±4.9 | b.d. | b.d. | 40±20 | 113±103 |
| **+ IL-Ra basolateral** | 1.4±1.5 | 21.2±18.8 | 15.9±14.8 | 19.3±18.2 | 1.0±0.5 | 5.9±2.9 | 27.2±13.9 | 47.7±20.6 | b.d. | 0.7±0.6 | b.d. | 8.5±8.9 | b.d. | b.d. | 22±19 | 66±27 |
| **+ IL-1Ra apical and basolateral** | 1.2±1.3 | 14.6±11.9 | 14.2±13.5 | 15.6±13.6 | 0.9±0.5 | 3.1±1.1 | 24.0±10.1 | 34.9±9.2 | b.d. | 0.8±6 | b.d. | 7.3±6.9 | b.d. | b.d. | 18±16 | 62±36 |

Table 2. Fibroblast and HBEC monocultures were stimulated with IL-1α either apically (10ng/ml), basolaterally (1ng/ml) or in combination, or with poly(I:C) (1µg/ml) as a positive control. After 24h, cell-free supernatants were assayed for IL-6 and CXCL8 by ELISA. Results are means ± SD, n=4-5. Raw data for figure 5A-D; Effect of IL-1α stimulation on IL-6 and CXCL8 release from fibroblast and HBEC monocultures.

|  | **IL-6 (ng/ml)** | | | | **CXCL8 (ng/ml)** | | | |
| --- | --- | --- | --- | --- | --- | --- | --- | --- |
|  | **Apical** | | **Basolateral** | | **Apical** | | **Basolateral** | |
|  | **Fibroblast** | **Epithelial** | **Fibroblast** | **Epithelial** | **Fibroblast** | **Epithelial** | **Fibroblast** | **Epithelial** |
| **control** | 0.4±0.4 | 0.4±0.2 | 0.7±0.7 | 0.6±0.3 | 0.3±0.2 | 0.8±0.2 | 0.3±0.1 | 1.4±0.3 |
| **dsRNA** | 0.6±0.6 | 6.1±3.7 | 0.9±0.8 | 1.9±1.1 | 0.5±0.4 | 5.3±0.8 | 0.4±0.2 | 2.6±0.2 |
| **IL-1α** | 49.8±42.4 | 0.4±0.2 | 72.5±58.8 | 0.7±0.3 | 146.6±113.7 | 1.1±0.2 | 137.1±125.6 | 1.7±0.4 |
